# Genome-wide SNPs lead to strong signals of geographic structure and relatedness patterns in the major arbovirus vector, *Aedes aegypti*

**DOI:** 10.1186/1471-2164-15-275

**Published:** 2014-04-11

**Authors:** Gordana Rašić, Igor Filipović, Andrew R Weeks, Ary A Hoffmann

**Affiliations:** 1 Pest and Disease Vector Group, Department of Genetics, The University of Melbourne, Victoria 3010, Australia

**Keywords:** *Aedes aegypti*, Restriction-site associated DNA sequencing, *In silico* genome digestion, Fastq file demultiplexing, Genome-wide single nucleotide polymorphisms, Mosquito population genomics

## Abstract

**Background:**

Genetic markers are widely used to understand the biology and population dynamics of disease vectors, but often markers are limited in the resolution they provide. In particular, the delineation of population structure, fine scale movement and patterns of relatedness are often obscured unless numerous markers are available. To address this issue in the major arbovirus vector, the yellow fever mosquito (*Aedes aegypti*), we used double digest Restriction-site Associated DNA (ddRAD) sequencing for the discovery of genome-wide single nucleotide polymorphisms (SNPs). We aimed to characterize the new SNP set and to test the resolution against previously described microsatellite markers in detecting broad and fine-scale genetic patterns in *Ae. aegypti*.

**Results:**

We developed bioinformatics tools that support the customization of restriction enzyme-based protocols for SNP discovery. We showed that our approach for RAD library construction achieves unbiased genome representation that reflects true evolutionary processes. In *Ae. aegypti* samples from three continents we identified more than 18,000 putative SNPs. They were widely distributed across the three *Ae. aegypti* chromosomes, with 47.9% found in intergenic regions and 17.8% in exons of over 2,300 genes. Pattern of their imputed effects in ORFs and UTRs were consistent with those found in a recent transcriptome study. We demonstrated that individual mosquitoes from Indonesia, Australia, Vietnam and Brazil can be assigned with a very high degree of confidence to their region of origin using a large SNP panel. We also showed that familial relatedness of samples from a 0.4 km^2^ area could be confidently established with a subset of SNPs.

**Conclusions:**

Using a cost-effective customized RAD sequencing approach supported by our bioinformatics tools, we characterized over 18,000 SNPs in field samples of the dengue fever mosquito *Ae. aegypti*. The variants were annotated and positioned onto the three *Ae. aegypti* chromosomes. The new SNP set provided much greater resolution in detecting population structure and estimating fine-scale relatedness than a set of polymorphic microsatellites. RAD-based markers demonstrate great potential to advance our understanding of mosquito population processes, critical for implementing new control measures against this major disease vector.

## Background

The yellow fever mosquito, *Aedes aegypti* (Culicidae, Diptera), is the major vector of human arboviruses. Because of its tight connection to humans and its distribution in most sub-tropical and tropical regions, this insect causes a substantial burden on global public health [1]. The development of anti-viral vaccines has been largely unsuccessful and has therefore shifted focus in disease prevention back to the control of *Ae. aegypti* populations [2]. Understanding basic ecological and microevolutionary processes in mosquito populations is necessary for the efficient implementation of many control measures. For example, knowledge of the rates of ongoing gene flow, fine scale mosquito movement and adaptive genomic changes are important for predicting the spread of *Wolbachia* infection [3,4] or insecticide resistance [5].

Ecological processes can often be inferred indirectly from observed genetic patterns [6] and this has led to interest in developing molecular markers for population studies in *Ae. aegypti.* In the last decade, mitochondrial DNA (mtDNA) sequences have commonly been used for *Ae. aegypti* population genetic studies [7]. However, mtDNA markers are best suited for analyses of historical processes at larger geographic scales, as they can only provide information on long-term accumulated effects of female dispersal due to their moderate mutation rate and maternal mode of inheritance [8]. Furthermore, the recent discovery of mtDNA pseudogenes (Numts) in the nuclear genome of *Ae. aegypti* limits the reliability of inferences from mtDNA [9]. Fast-mutating, highly variable nuclear markers such as microsatellites enable efficient analyses of fine-scale contemporary processes [8]. Close association with repetitive genetic elements and lower polymorphism has hindered the development of a robust set of microsatellite markers in *Ae. aegypti* until the complete genome sequence became available [10-12]. Population genetic structure of this vector has since been usually ascertained with up to 12 microsatellite loci [13], that were sometimes supplemented with a few single nucleotide polymorphism (SNP) markers [14] or Exon-Primed Intron Crossing (EPIC) markers [15-17].

Based on these marker systems, some details of *Ae. aegypti* movement and gene flow patterns have been inferred. Studies have investigated genetic structure across different spatial scales [7,13], and some have also included a temporal component [15-17]. Despite reports of limited active dispersal of this mosquito [18], a high level of gene flow has often been found even at broader regional and continental scales [15,19]. Unexpected structure may be explained by passive, human-mediated dispersal of mosquito eggs, larvae and adults coupled with low migration rates [7,13,20]. However, methodological issues associated with the available genetic markers [6,8] remain a major obstacle in using these markers to understand population movement patterns and microspatial scale structuring across time.

Next generation sequencing provides an opportunity to generate SNP markers at a genome-wide level even for non-model species. A mere 50 bi-allelic SNPs can provide the same resolution as 20 highly variable microsatellites for distinguishing closely related individuals [21]. A large panel of SNPs should therefore detect weak genetic structure caused by recent ecological and evolutionary processes [8] and provide reliable inferences of demographic history [22]. Genome-wide SNPs would thus be ideal for determining patterns of dispersal, gene flow and genetic structure at all spatial scales for populations of *Ae. aegypti*.

An analysis of genome-wide sequence variation *via* whole genome sequencing is still prohibitively expensive for *Ae. aegypti*, given its large genome size (1.3 Gbp; [23]). A cost-effective alternative is to sequence a fraction of the genome through Restriction-site Associated DNA sequencing (RADseq) [24,25]. RAD sequencing has been successfully used for the discovery of thousands of markers in yeast, plants and animals [26,27]. RAD loci are DNA fragments adjacent to the cut site of a particular restriction enzyme, or a combination of two enzymes [28]. Choice of restriction enzyme(s) (e.g. frequent or infrequent cutters) and the fragment size selection window can be used to optimize the number of tags, capturing a certain proportion of the genome for SNP detection and comparison across multiple individuals or populations.

Recently, Brown et al. [29] used 1,503 SNPs generated with RAD sequencing to test the hypothesis about global invasion and domestication of *Aedes aegypti.* Here, we developed bioinformatics tools that support the customization of restriction-enzyme-based protocols and used them to discover over 18,000 SNPs in *Ae. aegypti* collected on three continents. We have positioned these SNPs onto the newly assembled *Ae. aegypti* reference genome [30] and provided their annotation and prediction of effects. We tested the resolution of these new markers against a set of previously isolated microsatellites in delineating neotropical populations and establishing relatedness patterns at a fine spatial scale. Overall, we demonstrated the high potential of genome-wide SNP markers to advance our understanding of mosquito population dynamics which is crucial for improving the control measures against this major disease vector.

## Results and discussion

### Bioinformatics tools: *DDsilico* and *DDemux*

We developed *DDsilico* as a memory efficient program written in C language for *in silico* genome digestion with a single restriction enzyme or a combination of enzymes. This program calculates the number of potential RAD loci from the available genome sequence (see Additional file 1). *DDsilico* can be used for any restriction-enzyme-based protocol and is particularly suitable for customizing the double digest (dd)RADseq protocol [28] because it separates fragments that have different overhangs on two ends (i.e. potential double digest RAD loci) from non-usable fragments. We tested the performance of *DDsilico* by comparing results with the Bioanalyzer profiles of empirical digestions. DNA of the transformation vector Stinger GFP was digested with restriction enzymes *NlaIII* and *MluCI* (NEB). Observed and expected fragment profiles were highly concordant for sizes over 150 bp (see Additional file 2). Due to the purification of digestion reactions required for the Bioanalyzer run, fragments smaller than 150 bp were only partially retained with the paramagnetic bead solution.

To estimate the number of potential ddRAD tags in the *Ae. aegypti* genome, we performed *in silico* double digestion with various combinations of restriction enzymes. Distinguishing between usable fragments is a feature of *DDsilico*, as some double digestions produce numerous fragments in the desirable size range (100–500 bp), but only a small proportion constitutes potential ddRAD loci (see Additional file 3). Based on *in silico* results, we chose two frequent cutter enzymes (*NlaIII* and *MluCI*) for our ddRAD library construction. The ratio between the number of loci in the *Ae. aegypti* catalog (574,715) and the number of loci predicted with *DDsilico* (641,234) was 89.6%.

*DDsilico* can be used for any method that utilizes restriction enzyme(s) to create libraries with a reduced genome representation, such as Genotyping-by-sequencing (GBS) [31,32], Reduced-representation bi-sulfate sequencing (RRBS) [33], ezRAD [34] or RESTseq [35]. Simulating genome digestions enables researchers to choose optimal enzymes for specific species and research questions. For example, using rare cutting enzymes allows for higher level of sample multiplexing but can result in a non-uniform complexity reduction and biased distribution of sequenced fragments [35], leading to inaccurate estimation of population parameters or average methylation levels [33].

We also developed *DDemux* as a demultiplexer of fastq files under various barcoding schemes (see Additional file 4). Our program is memory efficient, developed in C language and easy to execute on Windows and Ubuntu platforms. It supports samples labelled with one or two barcodes that can vary in length. *DDemux* can use barcode sequences found in P1 and/or P2 reads, and in the Index reads (Illumina sequencing platforms). In our library, each sample was defined by a combination of variable length barcodes at the 5’ and 3’ ends (found in P1 and P2 reads). Such a combinatorial scheme reduces the cost for generating adapters with unique barcodes, while the varying length of barcodes increases library diversity at the 5’ and 3’ ends. This is particularly useful for ddRAD and other non-shearing-based libraries, where paired end (P2) reads can be used for SNP discovery but lack any diversity in the initial sequencing cycles when barcodes are absent.

**Figure 1:**
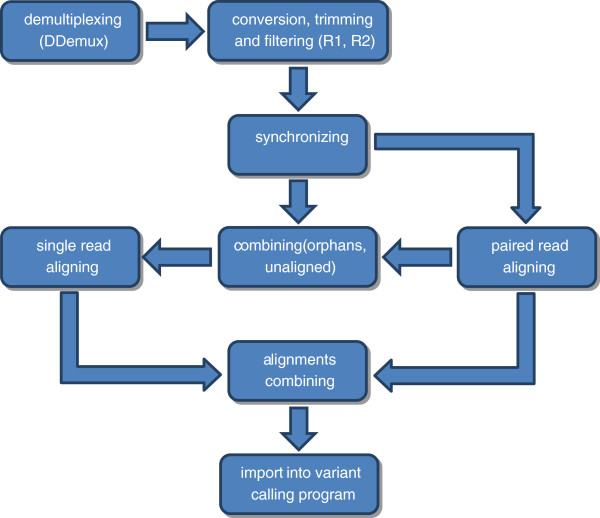
**Schematic representation of the customized pipeline for automated sequence processing.** After demultiplexing using *DDemux*, quality scores of individually sorted raw sequencing reads are converted to Sanger format. Reads are then filtered and trimmed according to the given parameters. Retained reads are then synchronyzed as pairs with identical coordinates, and unpaired reads sorted as orphans. Paired reads are aligned to the reference genome using Bowtie [36]. Unaligned and orphan reads are then joined, and user can choose to perform single read alignment. Finally, all aligned reads are joined and are ready for a variant calling pipeline.

We developed a customized pipeline that takes individually sorted raw sequences and outputs files ready for SNP calling (Figure 1, see Additional file 5). It automates standard processes for read quality control and aligning to a reference genome. Following demultiplexing, sequence quality scores are automatically converted into the Sanger format. This ensures standardization of quality scores for data generated by sequencing machines with different QC set-ups. Single (P1) and paired (P2) reads are then filtered and trimmed according to the chosen Phred score with FASTX-Toolkit (http://hannonlab.cshl.edu/fastx_toolkit/). P1 and P2 reads are then matched, leaving the unpaired reads as orphans. Paired reads are first aligned to the reference genome using Bowtie [36] according to the selected parameters. User can choose to also perform single-end alignment using joined orphans and all unaligned paired reads. In the final step, all aligned output files are merged per individual and are ready for downstream analyses.

### Discovery and characterization of SNPs in *Ae. aegypti*

Prior to any quality filtering, the average number of reads per individual was 3.31 million, ranging between 1.67 and 5.99 million reads. Quality filtering removed on average 5.5% of reads, retaining sequences with a mean quality score of 38 and a GC content of 41%. On average, 72.7% high quality reads aligned uniquely to the *Ae. aegypti* reference genome. With a minimum depth of five reads per individual, aligned sequences formed 574,715 RAD loci in the catalogue created with the *Stacks* pipeline for SNP discovery [37].

We further filtered loci that were present in at least 75% of individuals and retained 13,591 loci with an average 12× read depth (Table 1). 9,611 loci (70.72%) were polymorphic in at least one geographic sample, giving 18,147 putative biallelic SNPs (see Additional file 6). When we applied the same filtering criterion to each of the four samples, between 6,877 and 8,755 SNPs per sample were obtained. Number of private variants was the largest in the collection from Brazil (2,511) and the lowest from Vietnam (1,200). Average minor allele frequency per sample was between 0.187 (Vietnam) and 0.211 (Brazil) (Table 1).

**Table 1:** Summary statistics for filtered RAD loci

	***n***	**T**	**% pol**	**SNP**	**Private**	***P***	***H***_**O**_	***H***_**E**_	***F***_**IS**_	***π***	***N***_**e**_
Br	17	24273	23.64	8755	2511	0.800	0.265	0.282	0.079	0.0014	23083
Au	17	25019	21.68	8026	1310	0.810	0.234	0.268	0.120	0.0012	19479
In	13	25002	20.87	7757	1423	0.809	0.253	0.270	0.083	0.0012	19230
Vi	15	22333	20.61	6877	1200	0.815	0.243	0.261	0.079	0.0011	18401
All	62	13591	70.72	18147							

*n* - the number of analyzed individuals from Brazil (Br), Australia (Au), Indonesia (In) and Vietnam (Vi); T - the number of RAD loci; % pol - percentage of polymorphic loci; SNP - total number of SNPs; private - the number of private SNPs; *P* - average frequency of the more common allele; *H*_O_, *H*_E_ – observed and expected heterozygosity at polymorphic sites; *F*_IS_ - fixation index across polymorphic sites; *π* – average nucleotide diversity (calculated across polymorphic and non-variant sites); *N*_e_ – lower range effective population size (for *μ* = 10^-8^ per site per generation).

Most common sequencing errors of the Illumina machines are A↔C and G↔T transversions [38]. In our filtered data-set, two thirds of SNPs were transitions (transition:transversion ratio 1.62), suggesting a very small influence of sequencing error on our SNP calling. This is highly comparable to other RADseq studies that reported a ratio of 1.6 in the European eel [39], 1.65 in the eggplant [40] and 1.7 in the great tit [41].

SNPs from our catalog were distributed across 1,036 supercontigs that constitute 89% of the *Ae. aegypti* genome. Their number was significantly correlated with the size of supercontigs (Pearson *r* = 0.783, *p* < 0.01). Thanks to the recently improved assembly of the *A. aegypti* genome [30] we were able to position 66.4% of uncovered SNPs onto the three *A. aegypti* chromosomes: 2,423 SNPs on chromosome 1, 5,313 on chromosome 2 and 4,320 on chromosome 3 (see Additional file 6).

**Figure 2:**
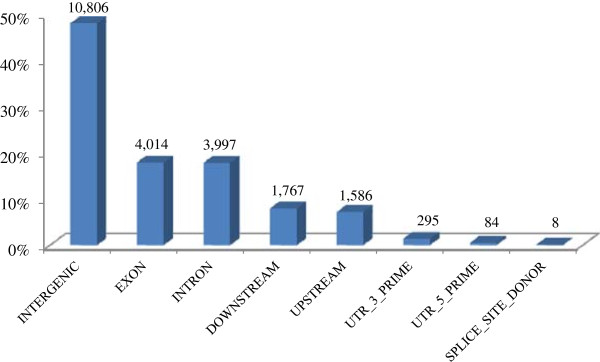
**Number of SNP effects by genomic region.** Variant effects were categorized using SNPEff [42] based on their position in the annotated *Aedes aegypti* genome. These include: introns, exons, untranslated region (5’ UTR and 3’ UTR), splice site or intergenic regions. “Upstream” is defined as a region 5 kilobase (kb) upstream of the most distal transcription start site and “downstream” as 5 kb downstream of the most distal polyA addition site [42]. Because some SNPs found between closely positioned genes were categorized as both upstream and downstream effects, the total number of effects was greater than the total number of SNPs.

**Figure 3:**
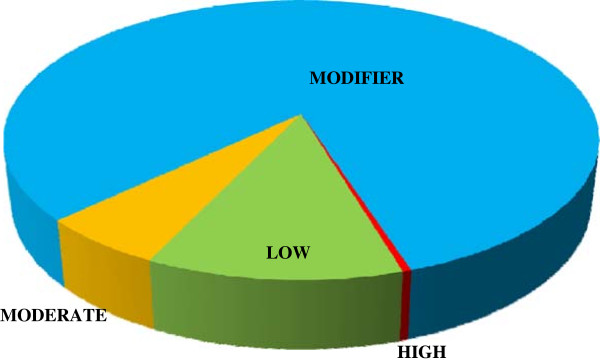
**Number of SNP effects by impact.** SNP effects were categorized by impact as high (affecting splice-sites, stop and start codons), moderate (non-synonymous), low (synonymous coding/start/stop, start gained), and modifier (upstream, downstream, intergenic, UTR).

We found 47.9% of SNPs in intergenic regions, 17.7% in introns and 17.8% in exons of 2,374 genes (Figure 2). They had very low occurrence in splice-site donor sequences and UTR 5’ region (0.035% and 0.301% respectively), and somewhat higher occurrence in UTR 3’ region (1.31%). Using the program SNPEff [42], we imputed the overall impact of all variants as largely modifying (82.1%), followed by low (11.7%), moderate (5.82%) and high impact (0.395%) (Figure 3, see Additional file 6).

Reduced representation libraries generally locate only around 2% of SNPs in the transcribed part of the genome [39], but they are generated using a rare cutting enzyme and random shearing. Here, we demonstrate the advantage of using two very frequent cutting enzymes that generate RAD loci evenly distributed across the genome. Other methods, such as RRSB, RESTseq or modified GBS, also utilize “common-cutters” to improve genome coverage and achieve unbiased distribution of sequenced loci [32,33,35].

Recently, Bonizzoni et al. [43] used RNAseq to analyze sequence variation in the transcriptome of three *Ae. aegypti* laboratory strains. They characterized over 130,000 SNPs in open-reading frames (ORFs) and untranslated regions (UTRs) of 4,492 genes. In our catalog, 19% of all SNPs were found within ORFs and UTRs of 2,374 genes. As in Bonizzoni et al., the most common SNPs were synonymous, 3’UTRs contained 4 times more SNPs than 5’UTRs, and SNPs that affect splice site donors, start and stop codons were very rare (total < 1%). We uncovered more non-synonymous changes than the mentioned study, likely because we analyzed pantropical *Ae. aegypti* populations that are very divergent from the laboratory reference strain LVP [13,29].

### Genetic diversity and effective population size

Average observed heterozygosity per variable SNP site was lower (0.23-0.26) than the expected heterozygosity (0.27-0.28) in all samples, with overall *F*_IS_ values ranging between 0.079 and 0.120 (Table 1). Microsatellite loci also showed lower than expected heterozygosity in all samples except for Vietnam, with *F*_IS_ ranging between 0.025 and 0.173 (see Additional file 7). Microsatellite loci were moderately polymorphic in all samples, with two to seven alleles per locus (average 3.75-4.50 alleles per locus).

Nucleotide diversity averaged over all SNP loci (π) was lower and comparable among samples (0.0011-0.0014, Table 1). Due to numerous low frequency alleles, this diversity index (equivalent to expected heterozygosity) was an order of magnitude lower than previously reported (e.g. 0.012 in [44]). Based on average nucleotide diversity, long term effective population sizes were estimated to range between 18,000 (for *μ* = 10^-8^ per site per generation) and 230,000 (for *μ* = 10^-9^ per site per generation) across four collections (Table 1). We note that SNPs may provide more biased estimates of long-term *N*_e_ than faster evolving markers such as microsatellites, because they are likely to be more affected by mutation-drift deviations [45]. However, our long term *N*_e_ based on the higher mutation rate (10^-8^ per site per generation) are concordant with the estimates of census population sizes at these collection sites. For example, Jeffery et al. [46] found that the number of *Ae. aegypti* adult females in Tri Nguyen village (Vietnam) could be as high as 26,431 individuals (95% CI 15,474-37,489), while we found the lower range of *N*_e_ to be 18,401 individuals (Table 1). Based on *Wolbachia-*infected *Ae. aegypti* releases in Gordonvale (Australia), a recent estimate for adult females within the release area was 7,261 individuals [47]. Assuming equal sex ratio, total census size in Gordonvale would be around 14,500 individuals, while our lower range *N*_e_ estimate was 19,479 individuals. Hence, we consider our SNP panel to be reflecting true evolutionary processes in *Ae. aegypti* populations.

### Using genome-wide SNPs to examine population processes

#### Broad-scale structuring

**Figure 4:**
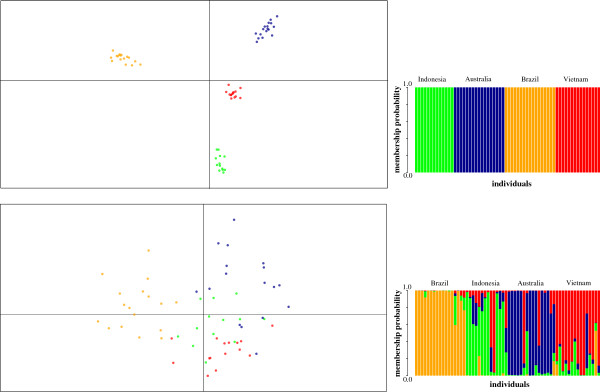
**DAPC scatterplots and membership probabilities for SNP and microsatellite data.** The scatterplots show the first two principal components of the DAPC of data generated with 18,147 SNP markers and 8 microsatellites. Geographic samples are represented in different colours (orange–Brazil, green–Indonesia, blue–Australia, red–Vietnam), with individuals shown as dots. Membership probabilities (in bar plots), interpreted as proximities of individuals to different clusters [48], show clear-cut separation of genetic groups for the SNP data and much weaker separation for the microsatellite data from Australia, Indonesia and Vietnam.

Our genome-wide SNP set demonstrated high power in resolving *Ae. aegypti* genetic structure at a broad spatial scale. Discriminant analysis of principal components (DAPC) with 18,147 SNPs revealed a clear separation of distinct genetic clusters, while the eight polymorphic microsatellites showed much less resolution in delineating Australian, Indonesian and Vietnamese samples (Figure 4). Ten principal components and three discriminant functions were retained in both analyses, conserving 41.4% of the variation for the SNP data and 73.7% of the variation for the microsatellite data. Membership probabilities, interpreted as proximities of individuals to different clusters [48], showed that genome-wide SNP markers achieved unambiguous separation of all groups.

Using 1,504 RAD-generated SNPs, Brown et al. [29] recently provided strong evidence for the African ancestry of domesticated *Ae. aegypti* that spread throughout the (sub)tropical New World and from there, relatively recently, invaded Southeast Asia and the Pacific. Their set of SNPs enabled clear separation of African and neotropical populations, but provided no resolution within the Southeast Asia/Pacific group [29]. Here we showed that strong separation between populations from Vietnam, Indonesia and Australia is achieved with more markers.

Pair-wise *F*_ST_ values were larger for SNP markers than for microsatellites (Table 2), which is expected given the intrinsic mathematical dependence of *F*_ST_ on heterozygosity, number of alleles and their frequency [49]. Despite our small sample sizes, degree of differentiation for microsatellite markers was high and comparable to previous studies. For example, Gordonvale and Tri Nguyen collections had a pair-wise *F*_ST_ value of 0.093 in Endersby et al. [15] and 0.092 in our study.

**Table 2:** **Estimates of pair-wise *F***_**ST**_[60]

	**Br**	**Au**	**In**	**Vi**
Br	-	0.146	0.108	0.177
Au	0.216	-	0.037	0.092
In	0.211	0.168	-	0.052
Vi	0.196	0.155	0.103	-

*F*_ST_ values computed over all loci are reported for microsatellite markers (above diagonal) and genome-wide SNPs (below diagonal).

#### Fine scale relatedness

**Figure 5:**
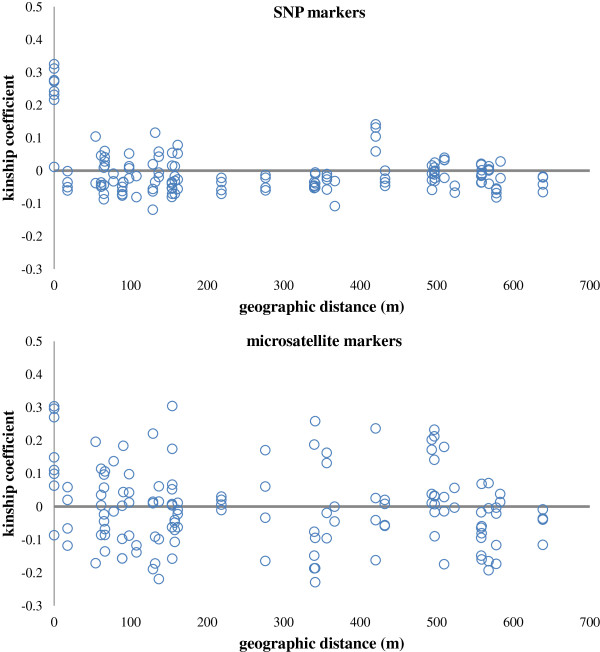
**Kinship coefficients across geographic distances (m).** Loiselle’s *k* was estimated using 934 SNP markers and 8 microsatellite markers for pairs of individuals sampled within a 0.4 km^2^ area on the Paqueta Island, Rio de Janeiro, Brazil.

934 SNPs showed remarkable power in distinguishing closely related individuals at a small spatial scale (Figure 5). Kinship coefficients (*k*) and maximum likelihood relatedness (*r*) were highly correlated (*r* = 0.884, *p* < 0.01) and all imputed relationships had a strong support (likelihood ratio test *p* < 0.001). Six full-sib pairs and one half-sib pair were detected in the same trap (geographic distance of zero, Figure 5), while three half-sib pairs were found 420 meters apart. Conversely, only 19% of all putative relationships obtained with microsatellite markers were statistically supported. Microsatellite kinship coefficients were higher across spatial distances, but their log likelihood was not significantly greater than the log likelihood of the alternative relationships. The only supported related pair (full-sib, likelihood ratio test *p* = 0.013) was found in the same trap (Figure 5).

The advantage of SNPs over microsatellites is increasingly reported in kinship and parentage analyses. Biallelic SNPs provide less information per locus, but this can be offset by their larger numbers. Unlike 17 microsatellites with low variability, a set of 960 SNPs ensured successful paternity and identity analysis in the European bison, *Bison bonasus*[50]. In the wild sockeye salmon (*Oncorhynchus nerka*), 80 SNPs outperformed 11 highly variable microsatellites in parentage and kinship assignment [51]. On the other hand, using too many SNPs can lead to a decrease in power due to information redundancy from non-independent markers [21], but selecting a subset of approximately independent SNPs (as we did, choosing SNPs located on different supercontigs) can overcome this issue.

With high-resolution genetic data, analyses of ecological processes in *Ae. aegypti* can shift from traditional ‘deme-based’ methods to new individual-based methods. Assignment tests and similar approaches provide more direct inferences of dispersal and contemporary gene flow, avoiding a number of problems associated with traditional indirect inferences [6,8]. Fine scale mosquito movement and egg-laying patterns become tractable with numerous markers that reveal genetic differences even between highly related individuals. Identification of barriers or corridors for dispersal of this mosquito can be undertaken *via* individual-based landscape genetic analyses [52].

We have demonstrated that RAD sequencing provides insight into various population patterns and processes, going from broad-scale structuring, effective population size to fine scale relatedness. RAD-based markers therefore have the great potential to advance our understanding of mosquito ecology and assist in developing appropriate measures of vector control at local and regional scales.

## Conclusions

Next-generation sequencing techniques such as RADseq provide an increasingly-affordable approach for generating numerous genetic markers to study disease vector populations. Here we developed a new set of bioinformatics tools that support the customization of restriction enzyme-based protocols for SNP discovery. We identified more than 18,000 putative SNPs in field samples of the major arbovirus vector *Ae. aegypti*. Our approach of using frequent cutting enzymes enabled unbiased sampling of genomic regions; we found 48% of variants in intergenic regions and 35% in exons and introns of over 2,300 genes. Their imputed effects in ORFs and UTRs were highly concordant with the effects found in the recent *Ae. aegypti* transcriptome sequencing study [43]. Our SNP set provided remarkable resolution in detecting broad-scale population structure and in estimating fine-scale relatedness. We demonstrated that a large SNP panel enables strong separation of *Ae. aegypti* populations even within a recently invaded neotropical region. Familial relatedness of samples collected from a small area could be confidently established with a subset of SNPs. RAD-based genetic data enables studies of basic mosquito ecology and evolution in a versatile manner and at an extremely fine resolution, facilitating targeted control measures designed for a particular local mosquito population.

## Methods

### Development of programs

We developed a memory-efficient program for *in silico* genome digestion and calculation of potential ddRAD loci numbers. *DDsilico* is written in C language and is compiled for execution on Windows and Linux operating systems (see Additional file 1). *DDsilico* reads the input file with a multiple-sequence fasta format, such as concatenated (super) contig or chromosome sequences. It outputs a text file with a distribution of fragments for a given bin size (1 - *n* bp), distinguishing between sequenceable fragments (different overhang on each end) and non-sequenceable fragments (the same overhang on both ends). Also, *DDsilico* outputs the sum of nucleotides within each bin, corresponding to the fluorescence intensity in the Bioanalyzer assay, for comparison with the empirical digestion.

We also created a program for demultiplexing fastq files that accommodates various indexing schemes. *DDemux* sorts reads for samples labelled with a single barcode or a combination of two barcodes that can vary in length (see Additional file 4). It was developed in C language and is compiled for execution on Windows and Ubuntu. *DDemux* has the capacity to sort reads for up to 125 samples in a single run.

### Sample collection

*Aedes aegypti* larvae and adults were collected from water containers, ovitraps and BG traps at four locations: Paqueta island, Rio de Janeiro (Brazil), Yogyakarta (Indonesia), Hon Mieu island (Vietnam) and Gordonvale (Australia). No specific field ethics approval is needed for the collection of wild mosquitoes in these areas. Verbal consent was obtained from residents at each location where collections occurred on private property. These locations were not on protected land and the field collections did not involve endangered or protected species. Thirteen to 17 individuals were analyzed from each location (Table 1). GPS location was recorded for each collected individual. Samples were stored in 80% ethanol at 4°C until processing. Genomic DNA was extracted using Qiagen DNA Blood and Tissue kit (Venlo, Limburg, NL), with the RNAse treatment step.

### Double digest RAD library preparation

100 ng of genomic DNA from each individual was digested in a 50 μL reaction with 100 units each of *NlaIII* and *MluCI* restriction enzymes (New England Biolabs, Beverly MA, USA), NEB Buffer 4, BSA and water for 3 hrs at 37°C, without a heat kill step. The digestion products were cleaned with 1.5× volume of Ampure XP™ paramagnetic beads (Beckman Coulter, Brea, CA) and ligated to the modified Illumina P1 and P2 adapters. We used a combinatorial indexing scheme, labelling each individual with a unique combination of P1 and P2 barcodes containing variable length barcodes to increase library diversity at 5’ and 3’ ends (see Additional file 8). This way, 16 P1 and three P2 adapters allow for multiplexing of 48 individuals, while achieving a 2.5-fold cost reduction for adapter generation. Forty μL ligation reactions were set up with 2 μL of 2 μM P1 and 6 μM P2 adapters, 1000 units of T4 ligase and 1× T4 buffer (New England Biolabs, Beverly MA, USA) and were incubated at 16°C overnight. Ligations were heat-inactivated at 65°C for 10 minutes and cooled down to a room temperature in a thermocycler at a rate of 1.5°C per 2 minutes. Adapter-ligated DNA fragments from all individuals were pooled and cleaned with 1.5× bead solution. Size selection of fragments between 300–450 bp was performed using a Pippin-Prep 2% gel cassette (Sage Sciences, Beverly, MA). Finally, 1 μL of the size selected DNA was used as a template in a 10 μL PCR reaction with 5 μL of the Phusion High Fidelity 2× Master mix (New England Biolabs, Beverly MA, USA) and 2 μL of 10 μM P1 and P2 primers [28]. PCR conditions were: 98°C for 30 s, 12 cycles of 98°C for 10 s, 60°C for 30 s, 72°C for 90 s, and the final elongation at 72°C for 5 min. Five such PCR reactions were pooled and cleaned with a 0.8× bead solution to make the final library. Two libraries were sequenced in two lanes of the Illumina HiSeq2000 platform to obtain 100 bp paired-end reads.

### Sequence processing and SNP calling

To automate the process, we wrote bash scripts that take individually sorted raw sequences and output files ready for SNP calling (Figure 1, see Additional file 5). Initially, sequence quality scores were automatically converted of into the Sanger format. Sequences were then filtered with FASTX-Toolkit, trimming the reads to 80 bp length and discarding all that have Phred score bellow 13. P1 and P2 reads were matched, and unpaired reads were sorted as orphans. Paired reads were aligned to the *Ae. aegypti* genome [53] using Bowtie version 0.12.7 [36]. Parameters for the un-gapped alignment included a maximum of three mismatches permitted in the seed, suppression of alignments if more than one reportable alignment exists, and a ‘try-hard’ option to find valid alignments. Orphans were then joined with all unaligned paired reads and single-end alignment was attempted. All aligned Bowtie output files were merged per individual and were imported into the *Stacks* pipeline.

A catalogue of RAD loci used for SNP discovery was created using the ref_map.pl pipeline in *Stacks* version 1.0 [37]. First, sequences aligned to the same genomic location were stacked together and merged to form loci. Here, only loci with a sequencing depth of five or more reads per individual were retained. SNPs at each locus were called using a maximum likelihood framework [54]. A catalogue was created of all possible loci and alleles and each individual was then matched against the catalogue. Finally, we used the program *Populations* in *Stacks* to process all the SNP data across individuals and calculate genome-wide measures of diversity, such as observed heterozygosity (*H*_O_), expected heterozygosity (*H*_E_) and nucleotide diversity (*π*). We also estimated the long-term effective population sizes (*N*_e_) using the nucleotide diversity averaged over all loci, where *π* = 4* *N*_e_*μ[55]. The mutation rate (*μ*) for SNPs is low, ranging between 10^-8^ and 10^-9^ per nucleotide site per generation [56].

To annotate and predict effects of filtered SNPs, we used SNPEff ver. 3.3 h [42] with default settings and *Ae. aegypti* gene set AaegL1.4 (http://www.vectorbase.org/organisms/aedes-aegypti/liverpool-lvp/AaegL1.4).

### Microsatellite genotyping

We also screened all individuals at eight microsatellite loci (AG5, BbH08, BbA10, AC1, 470AG1, M201, 69TGA1, BbB19) described previously [10-12]. Primers were ‘pig-tailed’ according to Brownstein et al. [57] and PCR products were directly labelled with fluorescent dye following the procedure by Blacket et al. [58]. Loci were separated into three multiplex 15 μL reactions. The reaction mix was prepared according to the microsatellite amplification procedure in the QIAGEN® Multiplex PCR Handbook and 0.5 ng of DNA. The cycling protocol included: the initial incubation step at 95°C for 15 minutes, 35 amplification cycles with 94°C for 30 s, 60°C for 90 s and 72°C for 60 s, followed by eight fluorescent labelling cycles with 94°C for 30 s, 53°C for 90 s and 72°C for 60 s, and final extension at 60°C for 30 minutes. Sizing of PCR products was done with Applied Biosystems 3730 DNA Analyser with 500 LIZ size standard. GeneMarkerV2.2.0 (Softgenetics, State College, PA) was used for allele scoring.

### Testing the SNP markers against microsatellites for detecting broad scale and fine-scale genetic patterns

We used Discriminant Analysis of Principal Components (DAPC) to identify and describe clusters of genetically related individuals implemented in the R package *adegenet* ver. 1.3-9.2 [48,59]. This multivariate method is suitable for analyzing large numbers of genome-wide SNPs, providing assignment of individuals to groups and a visual assessment of between-population differentiation. Because it does not rely on any particular population genetics model, DAPC is free of assumptions about Hardy-Weinberg equilibrium or linkage equilibrium [48].We therefore used a full set of 18,147 SNPs and eight microsatellites in this analysis. To avoid over-fitting of the discriminate functions, we retained ten principal components for both data sets. We also calculated Weir and Cockerhams’s *F*_ST_[60] in Genepop [61].

To explore the power of SNP and microsatellite markers to confidently assign relationships to pairs of individuals at a small spatial scale, we calculated Loiselle’s kinship coefficients *k*[62] for samples from Brazil in SPAGeDi [63]. First, in order to avoid strong linkage between SNPs, we made a subset of 934 markers by randomly sampling one SNP per supercontig. Then, as in Iacchei et al. [64], we considered Loiselle’s coefficients *k* to be between 0.25 and 0.375 for full-sibs and between 0.125 and 0.25 for half-sibs. A negative kinship coefficient indicates that a pair of individuals is less related than random pairs. We also calculated maximum likelihood relationship and used the likelihood ratio test with 1000 randomly simulated genotypes in ML-Relate [65].

### Availability of supporting data

Sequencing data are deposited at NCBI’s Sequence Read Archive (SRA) under the project accession number SRP040064 (http://www.ncbi.nlm.nih.gov/sra/?term=SRP040064). All other supporting data, including programs *DDsilico* and *DDemux*, are included as Additional files 1, 2, 3, 4, 5, 6, 7, 8.

## Competing interests

The authors declare that they have no competing interests.

## Authors’ contributions

AAH, ARW and GR conceived the study. GR designed and performed the RAD library constructions. IF and GR developed programs and performed bioinformatics analyses. GR analyzed data. GR and AAH drafted the manuscript. All authors have read and approved the final manuscript.

## Supplementary Material

Additional file 1 **The compressed archive contains *DDsilico *** **executables for Widows (ddsilico.exe) and Linux (ddsilico), the complete Stinger GFP sequence in fasta format (pstinger.fa) as a test input file, and README file (README.txt).** Click here for file

Additional file 2: Figure S2 Comparison of *DDsilico* and Bioanalyzer results for Stinger GFP. DNA of the transformation vector Stinger GFP was digested with restriction enzymes (*NlaIII* and *MluCI*) and compared with *DDsilico* results. Please note that the Bioanalyzer profile has two additional peaks at 35 bp and 10,380 bp that are the internal size standards for the High Sensitivity DNA chip (Agilent Technologies, Santa Clara, CA) (i.e. the two peaks are NOT part of the digested vector DNA). Also, Bioanalyzer peaks bellow 150 bp were only partially retained with the paramagnetic bead solution during the required purification step. Table S2. Fragment size distribution from the Bioanalyzer and *DDsilico* runs. Concordance between the two results is high, with only two very low intensity peaks (88 bp and 323 bp) present in the Bioanalyzer but absent in *DDsilico*. Click here for file

Additional file 3: Figure S3 *DDsilico* results for *Aedes aegypti* genome digested with various combinations of restriction enzymes. The x-axis represents fragment sizes (in base pairs), and the y-axis represent the number of fragments for a given size. Blue line depicts fragments that are not sequenceable (created by the same enzyme), while a red line depicts potential ddRAD loci. Distinguishing between amplifiable fragments is a useful *DDsilico* feature, as some double digestions produce numerous fragments in the desirable size range (100–500 bp), but only a small proportion constitutes potential ddRAD loci. Click here for file

Additional file 4 **The compressed archive contains *DDemux *** **executables for Widows (ddemux.exe) and Linux (ddemux), small Illumina fastq sequencing files (20000_R1.fq and 20000_R2.fq) as test input files, configuration file (config.txt) and README file (README.txt).** Click here for file

Additional file 5 **Compressed bash scripts that take individually sorted raw sequences and output files ready for SNP calling.** The compressed archive contains two scripts: preprocess_paired_reads.sh that also invokes sort_paired_reads.sh. preprocess_paired_reads.sh performs conversion of sequence quality score into the Sanger format, ensuring standardization of sequence data generated by various Illumina machines with different Q score set-ups. Sequences are then filtered with FASTX-Toolkit, trimmed to a desired reads length and filtered based on the Phred score. After these processes, reads are sorted and matched as pairs, while orphans are kept in separate files (for P1 and P2 reads). Reads are then uniquely aligned in Bowtie, as described in Materials and Methods. The final output file contains concatenated uniquely aligned reads for each sample ready for the SNP calling pipeline. Click here for file

Additional file 6 **Source SNP and genotyping data in VCF.** Compressed Variant calling format (VCF) file for a set of 18,147 SNPs filtered from the *Stacks* catalogue. Information field contains predicted SNP effects. Imputed individual genotypes from this file were used for the population genetic analyses. Additional file 6b is a tab delimited file with SNP positions in the improved chromosome assembly by Juneja et al. [30], where the last field (ID) corresponds to the ID field in VCF. Click here for file

Additional file 7: Table S6 Descriptive statistic for eight *Aedes aegypti m*icrosatellites. *N –* number of individuals screened for a particular microsatellite locus, *N*a – number of alleles per locus, *H*o – observed heterozygosity, *H*e – expected heterozygosity, *F*_IS_ – fixation index. Click here for file

Additional file 8 **Adapter and PCR primer sequences.** We have modified the adapter sequences from [28] by incorporating variable length barcodes on both P1 and P2 adapters to increase the sequence diversity at 5’ and 3’ ends and to create a cost-effective barcoding scheme. Click here for file
