# Supplementary material for: Genome-wide SNPs lead to strong signals of geographic structure and relatedness patterns in the major arbovirus vector, Aedes aegypti
Source: BMC Genomics. 2014 Apr 11;15:275. doi: 10.1186/1471-2164-15-275 (PMC4023594; doi:10.1186/1471-2164-15-275)
Supplement: Additional file 2: Figure S2 — Comparison of DDsilico and Bioanalyzer results for Stinger GFP. DNA of the transformation vector Stinger GFP was digested with restriction enzymes (NlaIII and MluCI) and compared with DDsilico results. Please note that the Bioanalyzer profile has two additional peaks at 35 bp and 10,380 bp that are the internal size standards for the High Sensitivity DNA chip (Agilent Technologies, Santa Clara, CA) (i.e. the two peaks are NOT part of the digested vector DNA). Also, Bioanalyzer peaks bellow 150 bp were only partially retained with the paramagnetic bead solution during the required purification step. Table S2. Fragment size distribution from the Bioanalyzer and DDsilico runs. Concordance between the two results is high, with only two very low intensity peaks (88 bp and 323 bp) present in the Bioanalyzer but absent in DDsilico. [file 1471-2164-15-275-S2.pdf]

Additional file 2. Figure S2.

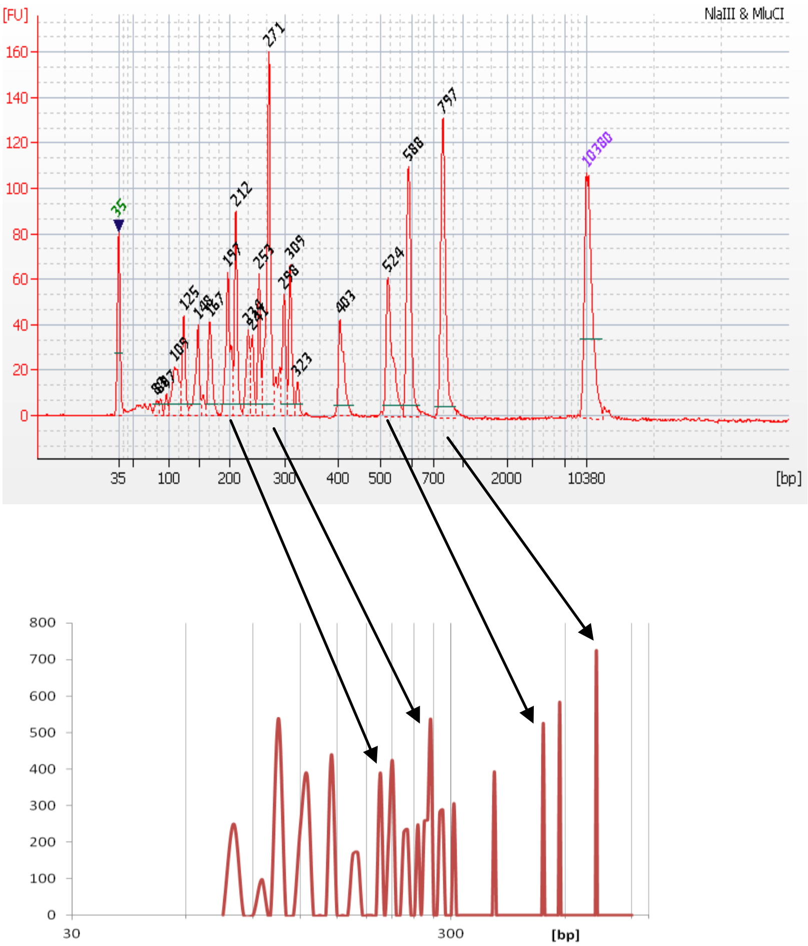

Table S2.

Fragment size (bp)		
Bioanalyzer	***DDsilico***	
83	present	80-85
88	**absent**	85-90
97	present	90-95
109	present	105-110
125	present	120-125
148	present	145-150
167	present	165-170
197	present	195-200
212	present	205-210
234	present	225-230
241	present	240-245
253	present	255-260
271	present	265-270
298	present	280-285
309	present	305-310
323	**absent**	320-325
403	present	390-395
524	present	520-525
588	present	580-585
797	present	725-730
