# Supplementary figures and images for: Genome-wide SNPs lead to strong signals of geographic structure and relatedness patterns in the major arbovirus vector, Aedes aegypti
Source: BMC Genomics. 2014 Apr 11;15:275. doi: 10.1186/1471-2164-15-275 (PMC4023594; doi:10.1186/1471-2164-15-275)

Additional file 3

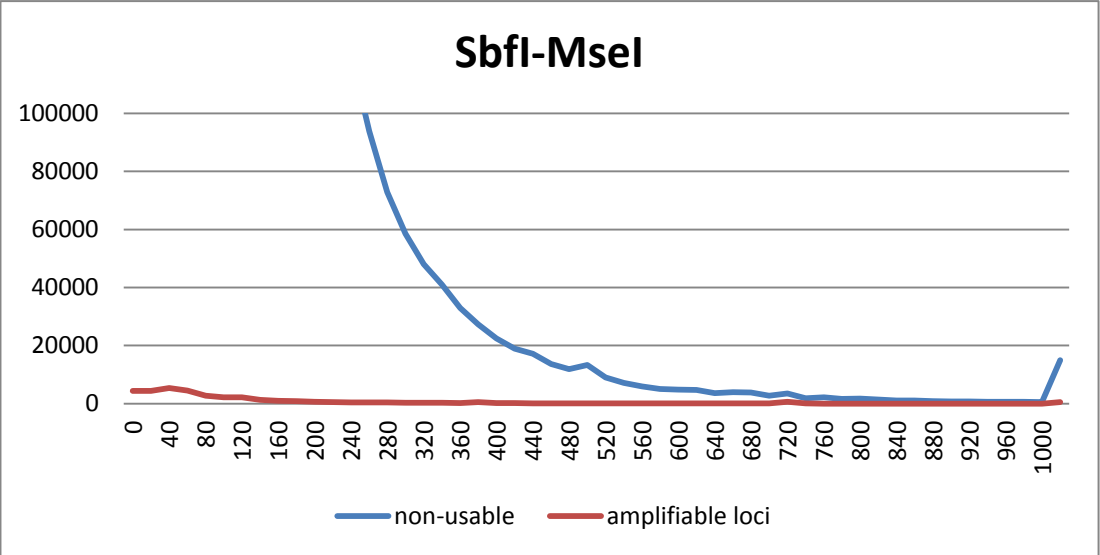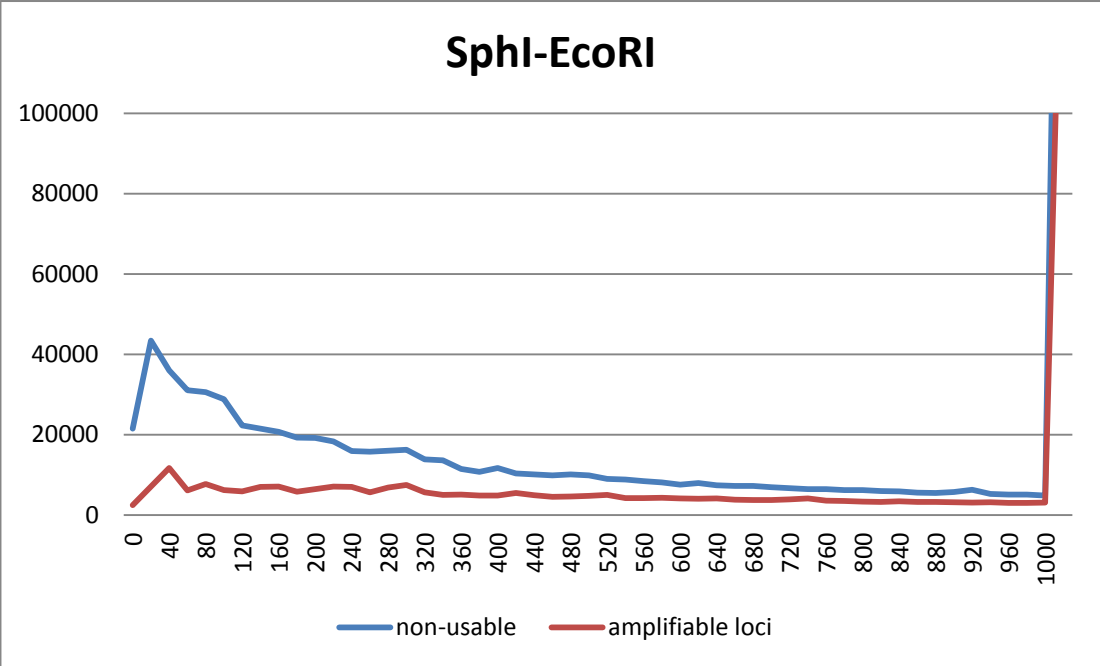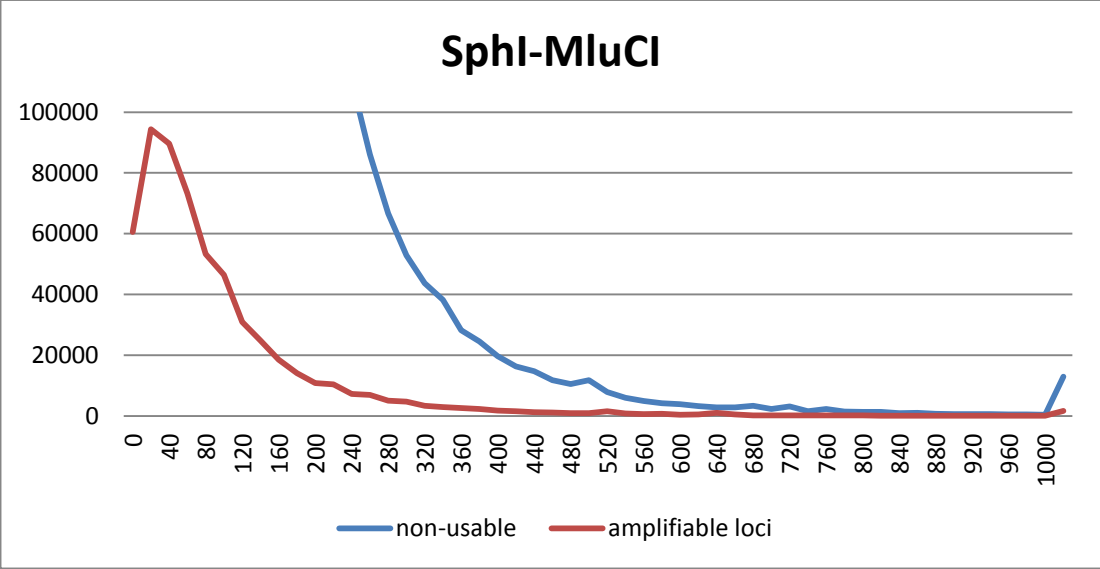

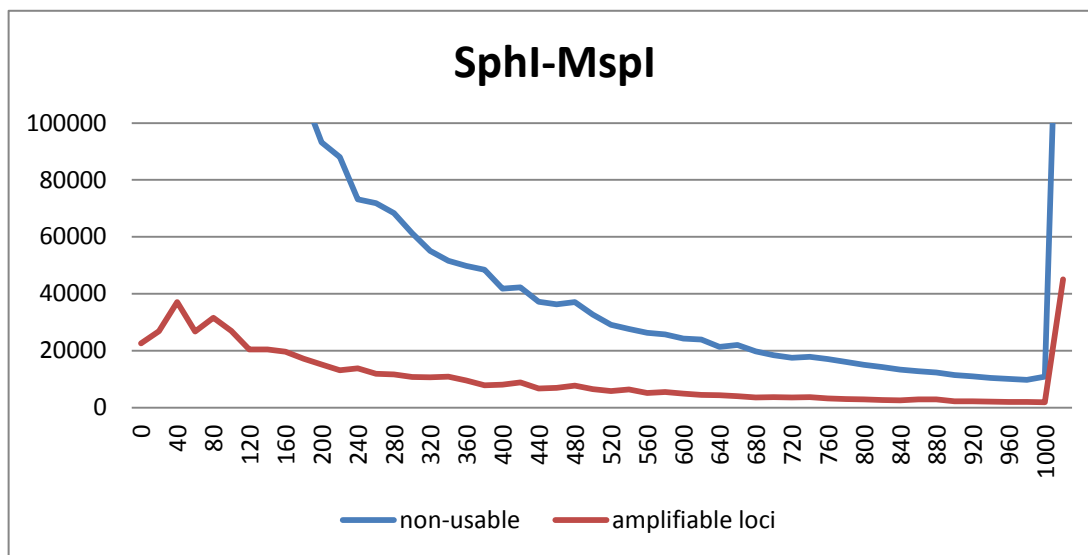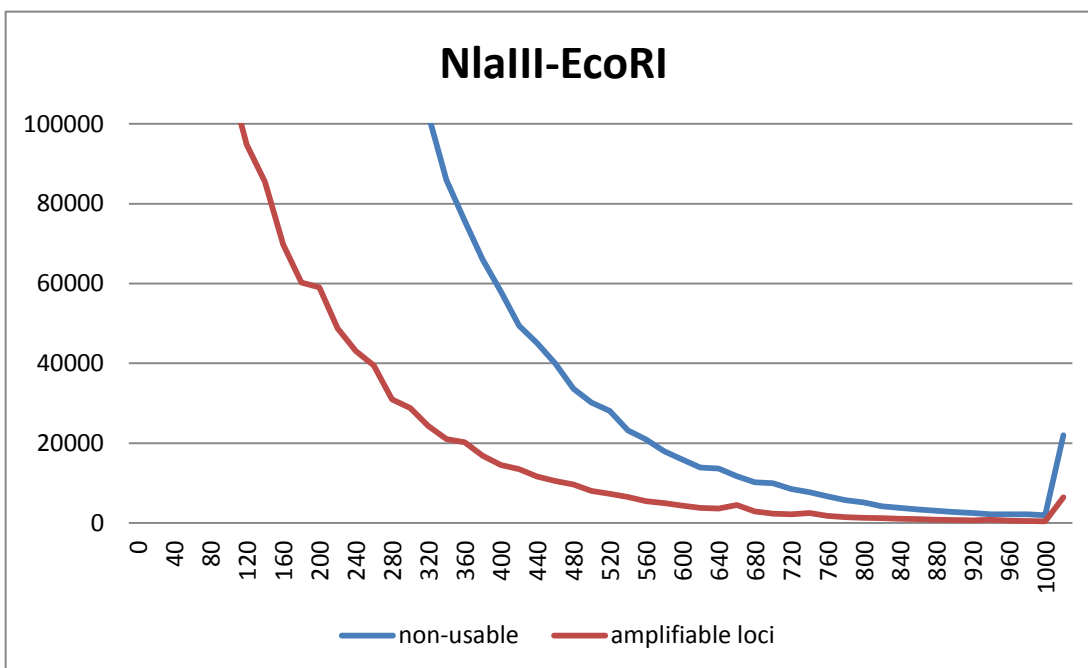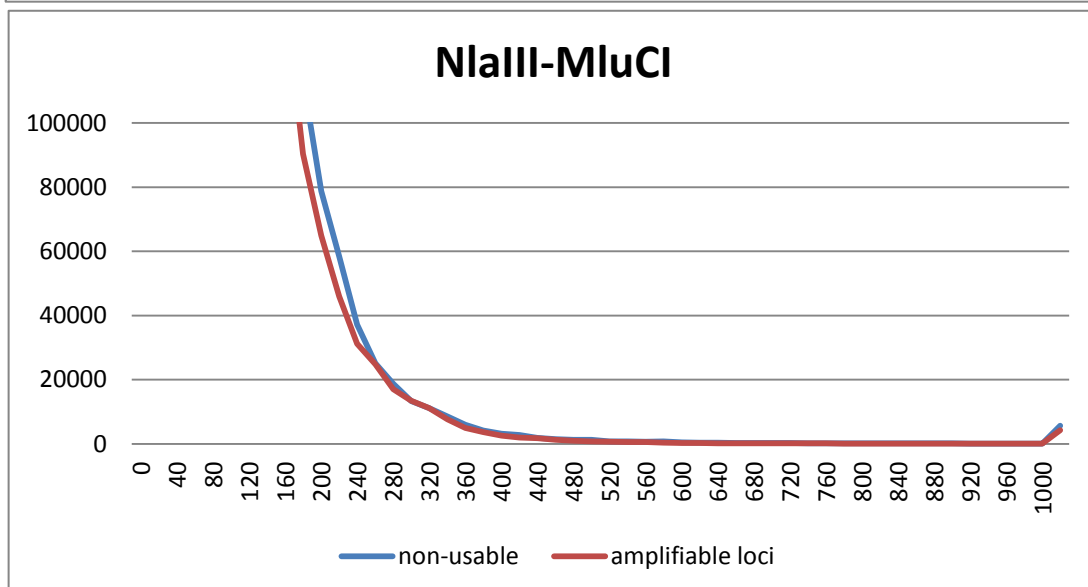

Supplement: Additional file 3: Figure S3 — DDsilico results for Aedes aegypti genome digested with various combinations of restriction enzymes. The x-axis represents fragment sizes (in base pairs), and the y-axis represent the number of fragments for a given size. Blue line depicts fragments that are not sequenceable (created by the same enzyme), while a red line depicts potential ddRAD loci. Distinguishing between amplifiable fragments is a useful DDsilico feature, as some double digestions produce numerous fragments in the desirable size range (100–500 bp), but only a small proportion constitutes potential ddRAD loci. [file 1471-2164-15-275-S3.pdf]
