# Supplementary material for: Genome-wide SNPs lead to strong signals of geographic structure and relatedness patterns in the major arbovirus vector, Aedes aegypti
Source: BMC Genomics. 2014 Apr 11;15:275. doi: 10.1186/1471-2164-15-275 (PMC4023594; doi:10.1186/1471-2164-15-275)
Supplement: Additional file 7: Table S6 — Descriptive statistic for eight Aedes aegypti microsatellites. N – number of individuals screened for a particular microsatellite locus, Na – number of alleles per locus, Ho – observed heterozygosity, He – expected heterozygosity, FIS – fixation index. [file 1471-2164-15-275-S7.pdf]

Additional file 6. Table S6.

Population		Ag5	AC1	BbH08	BbA10	BbB19	69TGA1	470AG1	M201
**Brazil**	***N***	17	17	17	17	14	17	15	17
	Na	4	4	4	7	3	3	3	2
	Ho	0.647	0.824	0.706	0.647	0.429	0.412	0.400	0.647
	He	0.663	0.727	0.692	0.763	0.559	0.457	0.464	0.500
	**u*He***	0.683	0.749	0.713	0.786	0.579	0.471	0.480	0.515
	F_{IS}	0.023	-0.133	-0.020	0.152	0.233	0.098	0.139	-0.294
**Indonesia**	***N***	13	13	13	13	13	13	12	13
	Na	6	6	4	6	4	5	3	2
	Ho	0.769	0.615	0.154	0.692	0.231	0.692	0.333	0.615
	He	0.740	0.790	0.544	0.757	0.553	0.698	0.448	0.426
	**u*He***	0.769	0.822	0.566	0.788	0.575	0.726	0.467	0.443
	F_{IS}	-0.040	0.221	0.717	0.086	0.583	0.008	0.256	-0.444
**Australia**	***N***	17	17	17	17	17	17	17	17
	Na	5	5	3	8	4	4	2	2
	Ho	0.765	0.529	0.471	0.588	0.235	0.412	0.412	0.941
	He	0.664	0.590	0.602	0.619	0.469	0.569	0.493	0.498
	**u*He***	0.684	0.608	0.620	0.638	0.483	0.586	0.508	0.513
	F_{IS}	-0.151	0.103	0.218	0.050	0.498	0.277	0.165	-0.889
**Vietnam**	***N***	15	15	15	15	15	15	14	15
	Na	5	3	2	5	4	5	4	2
	Ho	0.733	0.733	0.267	0.867	0.267	0.800	0.143	0.933
	He	0.656	0.487	0.231	0.669	0.464	0.753	0.452	0.498
	**u*He***	0.678	0.503	0.239	0.692	0.480	0.779	0.468	0.515
	F_{IS}	-0.119	-0.507	-0.154	-0.296	0.426	-0.062	0.684	-0.875
