# Supplementary material for: Genome-wide SNPs lead to strong signals of geographic structure and relatedness patterns in the major arbovirus vector, Aedes aegypti
Source: BMC Genomics. 2014 Apr 11;15:275. doi: 10.1186/1471-2164-15-275 (PMC4023594; doi:10.1186/1471-2164-15-275)
Supplement: Additional file 8 — Adapter and PCR primer sequences. We have modified the adapter sequences from [28] by incorporating variable length barcodes on both P1 and P2 adapters to increase the sequence diversity at 5’ and 3’ ends and to create a cost-effective barcoding scheme. [file 1471-2164-15-275-S8.pdf]

Additional file 7

adapter name	sequence
GCTGA_flex_P1.2	/5Phos/TCA GCA GAT CGG AAG AGC GTC GTG TAG GGA AAG AGT GT
GTAGT_flex_P1.2	/5Phos/ACT ACA GAT CGG AAG AGC GTC GTG TAG GGA AAG AGT GT
AACCA_flex_P1.2	/5Phos/TGG TTA GAT CGG AAG AGC GTC GTG TAG GGA AAG AGT GT
ACGCT_flex_P1.2	/5Phos/AGC GTA GAT CGG AAG AGC GTC GTG TAG GGA AAG AGT GT
CTTGG_flex_P1.2	/5Phos/CCA AGA GAT CGG AAG AGC GTC GTG TAG GGA AAG AGT GT
GGTTG_flex_P1.2	/5Phos/CAA CCA GAT CGG AAG AGC GTC GTG TAG GGA AAG AGT GT
TAGTA_flex_P1.2	/5Phos/TAC TAA GAT CGG AAG AGC GTC GTG TAG GGA AAG AGT GT
TTACC_flex_P1.2	/5Phos/GGT AAA GAT CGG AAG AGC GTC GTG TAG GGA AAG AGT GT
ACAGTG_flex_P1.2	/5Phos/CAC TGT AGA TCG GAA GAG CGT CGT GTA GGG AAA GAG TGT
TATAAT_flex_P1.2	/5Phos/ATT ATA AGA TCG GAA GAG CGT CGT GTA GGG AAA GAG TGT
AGTCAA_flex_P1.2	/5Phos/TTG ACT AGA TCG GAA GAG CGT CGT GTA GGG AAA GAG TGT
TAGTTT_flex_P1.2	/5Phos/AAA CTA AGA TCG GAA GAG CGT CGT GTA GGG AAA GAG TGT
GAGTGG_flex_P1.2	/5Phos/CCA CTC AGA TCG GAA GAG CGT CGT GTA GGG AAA GAG TGT
GCCAAT_flex_P1.2	/5Phos/ATT GGC AGA TCG GAA GAG CGT CGT GTA GGG AAA GAG TGT
CCAACA_flex_P1.2	/5Phos/TGT TGG AGA TCG GAA GAG CGT CGT GTA GGG AAA GAG TGT
CTGTAA_flex_P1.2	/5Phos/TTA CAG AGA TCG GAA GAG CGT CGT GTA GGG AAA GAG TGT
ATGTCAC_flex_P1.2	/5Phos/GTG ACA TAG ATC GGA AGA GCG TCG TGT AGG GAA AGA GTG T
GTGAAAC_flex_P1.2	/5Phos/GTT TCA CAG ATC GGA AGA GCG TCG TGT AGG GAA AGA GTG T
AGTTCCA_flex_P1.2	/5Phos/TGG AAC TAG ATC GGA AGA GCG TCG TGT AGG GAA AGA GTG T
TATAATG_flex_P1.2	/5Phos/CAT TAT AAG ATC GGA AGA GCG TCG TGT AGG GAA AGA GTG T
TACAGCA_flex_P1.2	/5Phos/TGC TGT AAG ATC GGA AGA GCG TCG TGT AGG GAA AGA GTG T
CAAAAGT_flex_P1.2	/5Phos/ACT TTT GAG ATC GGA AGA GCG TCG TGT AGG GAA AGA GTG T
CGATGTA_flex_P1.2	/5Phos/TAC ATC GAG ATC GGA AGA GCG TCG TGT AGG GAA AGA GTG T
GGTAGCA_flex_P1.2	/5Phos/TGC TAC CAG ATC GGA AGA GCG TCG TGT AGG GAA AGA GTG T
CAGT_flex_P2.1	/5Phos/AAT TAC TGA GAT CGG AAG AGC GAG AAC AA
CTCGG_flex_P2.1	/5Phos/AAT TCC GAG AGA TCG GAA GAG CGA GAA CAA
GTCGTC_flex_P2.1	/5Phos/AAT TGA CGA CAG ATC GGA AGA GCG AGA ACA A
GCTGA_flex_P1.1	ACA CTC TTT CCC TAC ACG ACG CTC TTC CGA TCT GCT GAC ATG
GTAGT_flex_P1.1	ACA CTC TTT CCC TAC ACG ACG CTC TTC CGA TCT GTA GTC ATG
AACCA_flex_P1.1	ACA CTC TTT CCC TAC ACG ACG CTC TTC CGA TCT AAC CAC ATG
ACGCT_flex_P1.1	ACA CTC TTT CCC TAC ACG ACG CTC TTC CGA TCT ACG CTC ATG
CTTGG_flex_P1.1	ACA CTC TTT CCC TAC ACG ACG CTC TTC CGA TCT CTT GGC ATG
GGTTG_flex_P1.1	ACA CTC TTT CCC TAC ACG ACG CTC TTC CGA TCT GGT TGC ATG
TAGTA_flex_P1.1	ACA CTC TTT CCC TAC ACG ACG CTC TTC CGA TCT TAG TAC ATG
TTACC_flex_P1.1	ACA CTC TTT CCC TAC ACG ACG CTC TTC CGA TCT TTA CCC ATG
ACAGTG_flex_P1.1	ACA CTC TTT CCC TAC ACG ACG CTC TTC CGA TCT ACA GTG CAT G
TATAAT_flex_P1.1	ACA CTC TTT CCC TAC ACG ACG CTC TTC CGA TCT TAT AAT CAT G
AGTCAA_flex_P1.1	ACA CTC TTT CCC TAC ACG ACG CTC TTC CGA TCT AGT CAA CAT G
TAGTTT_flex_P1.1	ACA CTC TTT CCC TAC ACG ACG CTC TTC CGA TCT TAG TTT CAT G
GAGTGG_flex_P1.1	ACA CTC TTT CCC TAC ACG ACG CTC TTC CGA TCT GAG TGG CAT G
GCCAAT_flex_P1.1	ACA CTC TTT CCC TAC ACG ACG CTC TTC CGA TCT GCC AAT CAT G
CCAACA_flex_P1.1	ACA CTC TTT CCC TAC ACG ACG CTC TTC CGA TCT CCA ACA CAT G
CTGTAA_flex_P1.1	ACA CTC TTT CCC TAC ACG ACG CTC TTC CGA TCT CTG TAA CAT G
ATGTCAC_flex_P1.1	ACA CTC TTT CCC TAC ACG ACG CTC TTC CGA TCT ATG TCA CCA TG
GTGAAAC_flex_P1.1	ACA CTC TTT CCC TAC ACG ACG CTC TTC CGA TCT GTG AAA CCA TG
AGTTCCA_flex_P1.1	ACA CTC TTT CCC TAC ACG ACG CTC TTC CGA TCT AGT TCC ACA TG
TATAATG_flex_P1.1	ACA CTC TTT CCC TAC ACG ACG CTC TTC CGA TCT TAT AAT GCA TG

adapter name	sequence
TACAGCA_flex_P1.1	ACA CTC TTT CCC TAC ACG ACG CTC TTC CGA TCT TAC AGC ACA TG
CAAAAGT_flex_P1.1	ACA CTC TTT CCC TAC ACG ACG CTC TTC CGA TCT CAA AAG TCA TG
CGATGTA_flex_P1.1	ACA CTC TTT CCC TAC ACG ACG CTC TTC CGA TCT CGA TGT ACA TG
GGTAGCA_flex_P1.1	ACA CTC TTT CCC TAC ACG ACG CTC TTC CGA TCT GGT AGC ACA TG
CAGT_flex_P2.2	GTG ACT GGA GTT CAG ACG TGT GCT CTT CCG ATC TCA GT
CTCGG_flex_P2.2	GTG ACT GGA GTT CAG ACG TGT GCT CTT CCG ATC TCT CGG
GTCGTC_flex_P2.2	GTG ACT GGA GTT CAG ACG TGT GCT CTT CCG ATC TGT CGT C

PCR primer name	sequence
PCR1	AAT GAT ACG GCG ACC ACC GAG ATC TAC ACT CTT TCC CTA CAC GAC G
PCR2	CAA GCA GAA GAC GGC ATA CGA GAT CGT GAT GTG ACT GGA GTT CAG ACG TGT GC
